# Pneumatosis cystoides intestinalis associated with etoposide in hematological malignancies: a case report and a literature review

**DOI:** 10.1186/s12876-022-02219-8

**Published:** 2022-03-28

**Authors:** Liqing Yang, Xi Zhong, Hao Yang, Qin Wu, Yuping Gong, Bo Wang

**Affiliations:** 1 Department of Hematology, West China Hospital, Sichuan University, Chengdu, 610041 Sichuan Province People’s Republic of China; 2 Department of Critical Care Medicine, West China Hospital, Sichuan University, Chengdu, 610041 Sichuan Province People’s Republic of China

**Keywords:** Pneumatosis cystoides intestinalis, Pneumoperitoneum, Intra-abdominal pressure, Etoposide, Hematological malignancy

## Abstract

Pneumatosis cystoides intestinalis (PCI) is a rare condition characterized by the presence of air collection within the subserosa and/or submucosa of the gastrointestinal wall. Due to the lack of specific symptoms, PCI is likely to be misdiagnosed or missed without the use of imaging techniques or gastrointestinal endoscopy. Here, we report a patient who complained of abdominal distention and constipation after chemotherapy for hematological malignancies, and was diagnosed with secondary PCI via computed tomography (CT) and exploratory laparotomy. Pneumoperitoneum was no longer observed after two weeks of conservative treatments. Notably, the possibility of intra-abdominal pressure (IAP) as a predictor for surgical intervention was proposed. Furthermore, we conducted a literature review on PCI after chemotherapy in hematological malignancies to raise awareness of etoposide-related PCI, while whether PCI could be identified as an adverse event of etoposide requires more evidence.

## Introduction

Pneumatosis cystoides intestinalis (PCI), first described by Du Vernoi in 1730, is characterized by the presence of air collection within the subserosa and/or submucosa of the gastrointestinal wall particularly in the ileum and colon [1, 2]. PCI is a rare condition that lacks accurate morbidity estimates due to the lack of specific symptoms, and should be suspected in the setting of pneumoperitoneum or free gas under the diaphragm without peritoneal irritation [3]. An increasing number of cases have been identified recently with the assistance of imaging examination and gastrointestinal endoscopy. The clinical manifestations of PCI vary from asymptomatic to lethal conditions, and common symptoms include abdominal pain, abdominal distention, nausea, vomiting, diarrhea and constipation [4, 5]. Primary PCI, which is relatively uncommon, is defined as an idiopathic disorder, and has been reported to be closely associated with chronic trichloroethylene exposure [6]. PCI secondary to various clinical conditions accounts for 85% of cases [7], including: (a) mechanical trauma: acute gastrointestinal diseases [8], and gastrointestinal carcinoma [9]; (b) autoimmune disorders: inflammatory bowel diseases (IBD) [10], and connective tissue diseases [11–13]; (c) infections: Clostridium difficile [14], and HIV [15, 16]; (d) pulmonary diseases: chronic obstructive pulmonary disease (COPD) [17]; (e) immunosuppressive states: during and after chemotherapy for malignant tumors [18, 19], and post hematopoietic stem cell transplantation (HSCT) [20]; and (f) drug induced: diabetes treated with alpha-glucosidase inhibitors (α-GI) [5]. Here, we present a case of PCI after etoposide-related chemotherapy for hematological malignancies that was initially misdiagnosed as gastrointestinal perforation with equivocal CT findings and underwent an exploratory laparotomy, confirming the diagnosis of PCI.

## Case presentation

A 14-year-old male patient was urgently sent to our hospital due to sudden dyspnea and disturbance of consciousness four hours earlier. He had complained about a week of vague body pain and two days of fever, progressively worsening manifesting as distinct abdominal distension and an absence of flatus one day before, with a small amount of stool being observed the day before.

Suffering from recurrent fever and noticeable enlargement of cervical lymph nodes, this pediatric patient was diagnosed with chronic active Epstein-Barr virus (CAEBV) infection a year earlier before and received long-term treatment with prednisone (40 mg/d). The parents were healthy and denied a history of familial cancers or genetic disorders. Herpes with rupture on the inner left thigh prompted the first visit to our hospital two months prior, when the patient had developed EBV-lymphoproliferative disease (EBV-LPD) merged with hemophagocytic syndrome (HPS). The ED regimen (etoposide 100 mg biw + dexamethasone 15 mg qd) was initiated three weeks earlier. Given the potential of lymphoma, tislelizumab, an inhibitor of programmed cell death-1 (PD-1), was provided at a dose of 100 mg two weeks earlier. The dose of primary regimen was reduced due to hyperpyrexia occurring five days later, which was presumed to be related to cytokine release syndrome (CRS) induced by the PD-1 inhibitor. The cumulative dose of etoposide was 425 mg. The patient did not complain of any abdominal discomfort in the week after using tislelizumab, and the stool was normal.

**Fig. 1:**
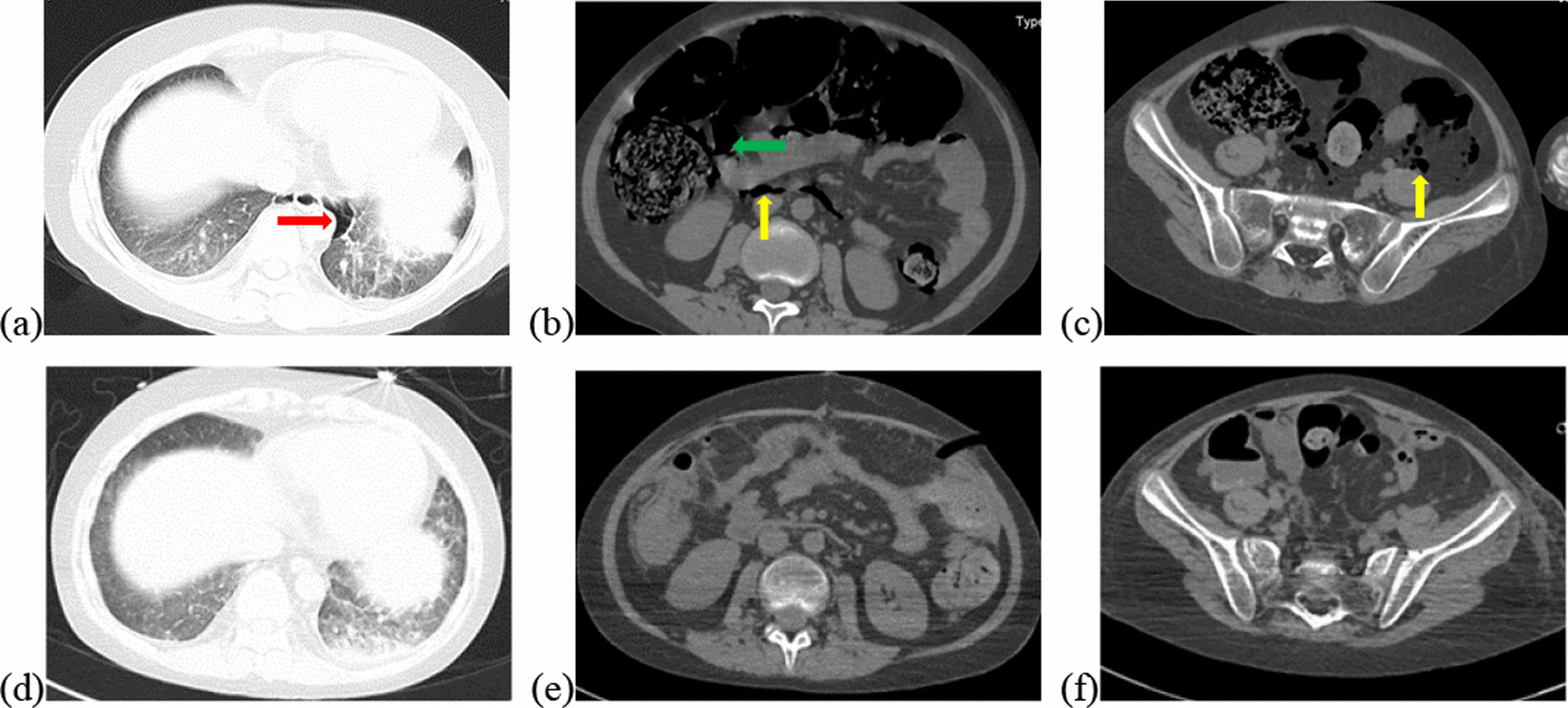
Initial abdominal CT and one week later. Initial abdominal CT scan showed massive free gas collected in the colon wall denoted by the green arrow (**b**). The gas could even be observed in the mediastinum in the lung view (**a**) denoted by the red arrow, retroperitoneal space in the abdomen (**b**) and pelvic cavities view (**c**) denoted by the yellow arrows. Free accumulated gas was gradually absorbed after one week of conservative treatments in the same view (**d**–**f**)

**Fig. 2:**
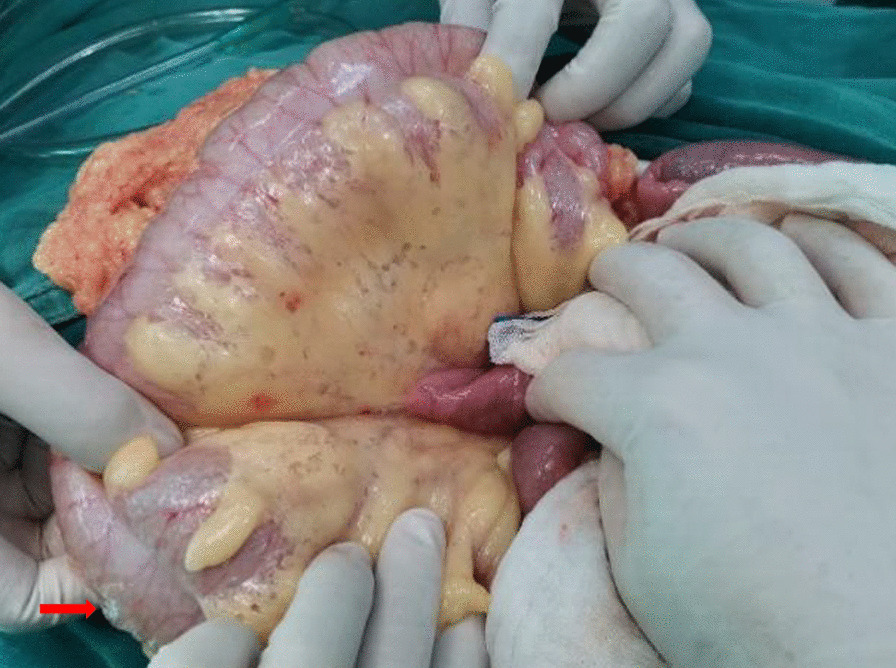
Intraoperative finding in exploratory laparotomy. Several bright bubbles could be observed within the colon wall with noteworthy crepitus. A bubble was denoted by the red arrow

**Fig. 3:**
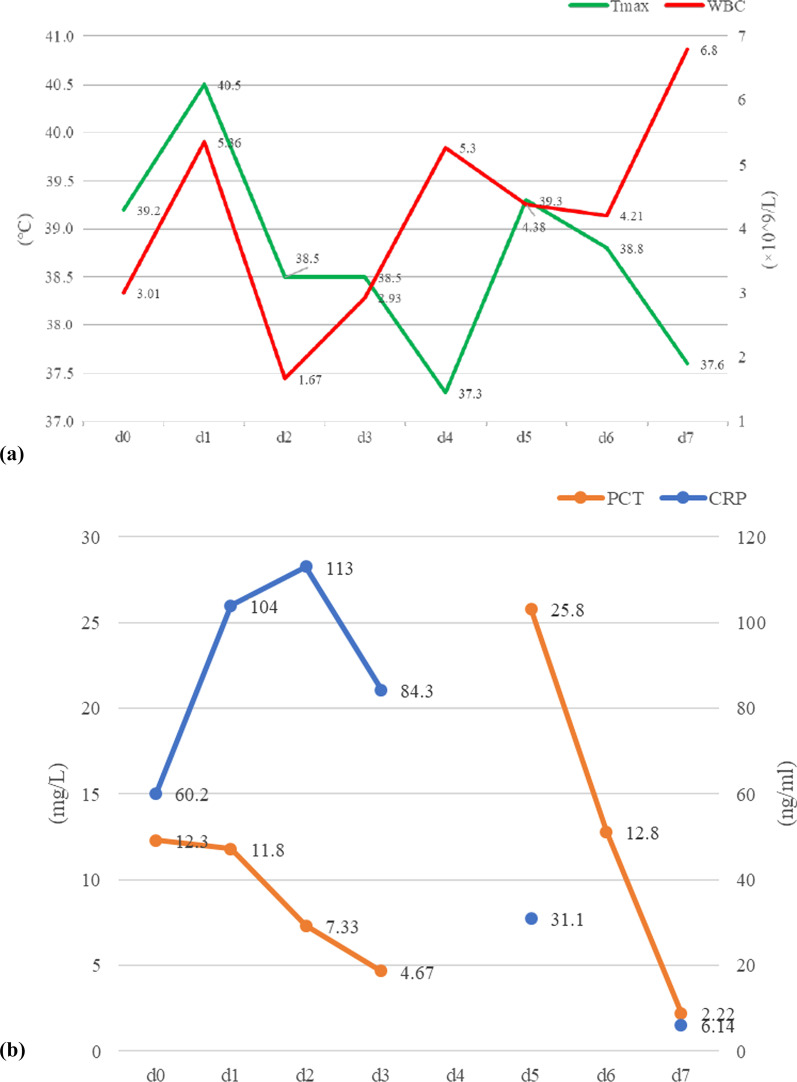
Observation of body temperature and inflammatory biomarkers in the following week (d1–d7) after the surgery. Abbreviation: Tmax = the highest body temperature in the day (the green line), *WBC* white blood cells (the red line), *PCT* procalcitonin (the orange line), *CRP* C reaction protein (the blue line)

**Fig. 4:**
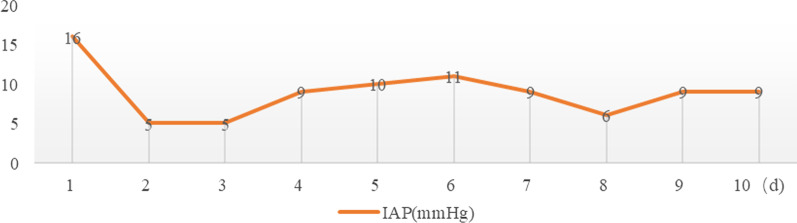
IAP was measured based on the trans-bladder technique in the first ten days

Vital signs at admission were as follows: T 38.4℃, P 190 bpm, R 25 bpm, BP 122/87 mmHg, and SpO_2_ 70%. An abdominal CT scan demonstrated that massive free gas was collecting within the colon wall (Fig. 1b), and even within the retroperitoneal space of the abdominal (Fig. 1b) and pelvic cavities (Fig. 1c), without enough evidence for peritonitis or seroperitoneum. Free air could be observed in the mediastinum in the lung view of chest CT (Fig. 1a) with slight pulmonary inflammation. Since we suspected gastrointestinal perforation, an exploratory laparotomy was performed immediately but no inflammation or gastrointestinal perforation was discovered. Intriguingly, gas accumulation was observed throughout the mesocolon wall with noteworthy crepitus (Fig. 2), confirming the diagnosis of PCI. The patient was admitted to the intensive care unit due to failure of postoperative tracheal extubation. Arterial blood gas analysis showed: pH 7.398, PaO_2_ 179.9 mmHg, PaCO_2_ 25.7 mmHg, lactic acid 2.3 mmol/L, and HCO_3_ 15.5 mmol/L. Subsequent auxiliary examinations supported the activation of EBV infection with an increased level of EBV-DNA (3.29 × 10^4^ copies/ml) and impairment of the immune system (CD3 counts 555 cell/ul, CD4 counts 123 cell/ul, CD8 counts 392 cell/ul). Furthermore, HPS was activated with the presentation of pancytopenia (HGB 68 g/L, WBC 3.01 × 10^9^/L, PLT 36 × 10^9^/L) and prominent elevation of ferritin (27,487 ng/ml) and IL-2R (1752 u/ml). Simultaneous liver function tests exhibited the following: AST 109 IU/L, ALT 60 IU/L, ALP 512 IU/L, and LDH 2479 IU/L. Body temperature and inflammatory biomarkers observed the following week are presented in Fig. 3. Prolonged fever was assumed to be associated with HPS since repeated secretion and serological cultures for etiological evidence of infection were inconclusive, and the determination for Clostridium difficile infection was not performed. Considering acute respiratory failure syndrome (ARDS) and latent intestinal infections, mechanical ventilation and antibiotic therapy were provided. In addition, nasogastric tube drainage and parenteral nutrition were both administered. Chemotherapy was interrupted and replaced by an injection of dexamethasone (7.5 mg/d) without additional adverse effects. Abdominal distention and constipation were gradually alleviated, and little free gas was observed in the bowel wall two weeks later (Fig. 1d–f). Furthermore, intra-abdominal pressure (IAP) was measured based on the trans-bladder technique in the first ten days (Fig. 4), which suggested an elevated IAP in the beginning, consistent with the significant abdominal distention.

## Search strategy and literature review

PCI is a benign phenomenon of pneumatosis intestinalis (PI), which is more frequently reported in adults and males according to a systematic analysis [21]. According to a literature review, PI in hematological disorders was frequently reported in debilitated patients after bone marrow transplantation, complicated with cutaneous or digestive graft versus host diseases (GVHD) in particular [22–26], while the significance of causative agents remains to be explored due to a limited number of cases. Hence, we conducted an advanced retrieval in the *PubMed* database up to December 2021 with a result of 2878 articles based on the following search strings: “pneumatosis cystoides intestinalis” OR “pneumatosis intestinalis” OR “pneumatosis”. Reviews and cases concerning nonhematologic disorders were excluded, and six studies [27–32] were filtered from the original 34 results based on the inclusion criteria: (1) clinical cases with a detailed description; (2) hematologic malignancy as the major diagnosis; and (3) treatment post chemotherapy without allo-HSCT. Twelve cases reported in the selected literature are listed in Table 1, where steroids (prednisolone/dexamethasone) and etoposide were used to treat eleven and three patients, respectively, and no records referred to tislelizumab-related therapy.

**Table 1:** Previous cases of PI/PCI post chemotherapy in hematologic malignancies

Case [Ref.]	Sex/age (yr)	Underlying disease	Chemotherapeutics	Clinical symptoms	Diagnosis/tools/location	Treatments
1 [27]	F/2.5	c-ALL	Prednisone, daunorubicin, vincristine, asparaginase, and intrathecal triple therapy	Lethargy abdominal distension	PCI/X-ray/colon	Antibiotics
2 [28]	M/55	Ph + CML	Nilotinib	Abdominal distension abdominal pain	PI/CT/small intestine	Oxygen therapy
3 [29]	M/31	T-ALL	Cyclophosphamide, mercaptopurine, cytosine arabinoside, and prednisone	Abdominal pain	PI/X-ray + CT/colon	Parenteral nutrition antibiotics
4 [29]	M/58	Lymphoma	BCNU, melphalan, and etoposide, cytosine arabinoside and dexamethasone	None	PI/X-ray + CT/colon	Parenteral nutrition antibiotics
5 [29]	F/64	Lymphoma	Cyclophosphamide, vincristine, doxorubicin, and prednisone	Abdominal pain fever, nausea, diarrhea	PI/CT/small intestine + colon	Parenteral nutrition antibiotics
6 [29]	F/49	SAA	Ciclosporine A, prednisone	Fever	PI/CT/enterocoelia	Parenteral nutrition antibiotics
7 [30]	M/18	B-ALL	Prednisolone, daunorubicin, vincristine, asparaginase, and intrathecal methotrexate	Abdominal distension anorexia	PI/X-ray + CT /colon + enterocoelia	Oxygen therapy antibiotics parenteral nutrition
8 [31]	F/33	APL	ATRA, idarubicin, dexamethasone	Abdominal pain nausea, diarrhea	PI/CT/small intestine + colon	Antibiotics
9 [32]	M/51	Lymphoma	Methotrexate, leukovorine rescue, mithoxantrone, cyclophosphamide, vincristine, prednisolone, and etoposide	Vague abdominal discomfort	PCI/X-ray/colon	Oxygen therapy antibiotics parenteral nutrition
10 [32]	F/42	AML-M1	Behenoyl cytosine arabinoside, daunorubicin, 6-mercaptopurine, and prednisone	Abdominal distension abdominal pain	PCI/X-ray/colon	Oxygen therapy
11 [32]	F/58	Lymphoma	Methotrexate, leukovorine rescue, mithoxantrone, cyclophosphamide, vincristine, prednisolone, and etoposide	Abdominal distension	PCI/X-ray/colon	Oxygen therapy parenteral nutrition
12 [32]	M/74	AML-M2	Cytosine arabinoside, prednisolone, etopside, and mitoxantrone	Abdominal distension	PCI/X-ray/colon	Oxygen therapy parenteral nutrition

*F* female, *M* male, *ALL* acute lymphocytic leukemia, *CML* chronic granulocytic leukemia, *SAA* severe aplastic anemia, *APL* acute promyelocytic leukemia, *AML* acute myeloblastic leukemia, *PI* pneumatosis intestinalis, *PCI* pneumatosis cystoides intestinalis, *ATRA* all-trans retinoic acid, *BCNU* carmustine

Although steroids have been proven to be significantly linked to the development of PCI [1, 33], our patient had received corticosteroid treatment for a long period even during PCI therapy. Therefore, further research on PCI/PI post chemotherapy based on etoposide and/or tislelizumab was conducted. As a novel monoclonal antibody in recent years, PD-1 inhibitors have shown great potential in hematologic malignancies, while reported side-effects concerning PI are lacking.

Six cases of PCI/PI associated with etoposide are described in Table 2 [29, 32, 34, 35]. Details including sex, age, underlying diseases, chemotherapy, causative agent, location, associated symptoms, diagnostic tool, treatments, outcome and time to recovery, were collected. The results suggested that abdominal distension was the most common symptom in etoposide-related PCI/PI. The majority of cases were diagnosed with X-ray (83.33%), and notably, the colon was the most frequent location (100%). Conservative treatments were effective and pneumoperitoneum subsided in no more than 30 days.

**Table 2:** Previous cases of PI/PCI post etoposide-based chemotherapy

Case [Ref.]	Sex/Age (yr)	Underlying disease	Chemotherapy	Causative agent, dose	Location	Diagnostic tools	Associated symptoms	Complications	Treatments	Outcome	Time to recovery
1 [29]	M/58	Lymphoma	NA	Etoposide, NA	Total colon	X-ray + CT	None	No	Parenteral nutrition antibiotics	Resolved	30 days
2 [32]	M/51	Lymphoma	MNCOP-V	Etoposide, 100 mg	Total colon	X-ray	Septicemia and abdominal discomfort	No	Oxygen therapy parental nutrition antibiotics	Resolved	NA
3 [32]	F/58	Lymphoma	MNCOP-V	Etoposide, 95 mg	Total colon + terminal ileum	X-ray	Slight abdominal distension	No	Oxygen therapy parental nutrition	Resolved	1 week
4 [32]	M/74	AML-M2	NA	Etoposide, NA	Total colon	X-ray	Abdominal distension	No	Oxygen therapy parental nutrition	Resolved	2 weeks
5 [34]	F/53	Breast cancer	Various	Etoposide, 50 mg qd	Total colon + rectum	X-ray + CT + colonoscopy	Severe abdominal distension with decreased flatus	No	Oxygen therapy antibiotics	Resolved	3 weeks
6 [35]	M/69	Small cell lung cancer	Carboplatin (d1) + etoposide (d1–3)	Etoposide, 100 mg	Sigmoid colon + retroperitoneum + posterior mediastinum	CT	Abdominal distension	No	Oxygen therapy antibiotics	Resolved	2 weeks

*M* male, *F* female, *AML* acute myeloid leukemia, *MNCOP-V* methotrexate with leucovorin rescue, mitoxantrone, cyclophosphamide, vincristine, prednisolone and etoposide, *NA* not available

## Discussion and conclusion

In this study, we presented a 14-year-old male patient who suffered from EBV-related hematological malignancies developed PCI after chemotherapy. It was reasonable to consider the potential of gastrointestinal perforation due to the massive amount of free gas in the abdominal cavity and even within the mediastinum, and the dramatically deteriorated conditions. PCI was confirmed in our patient with exploratory laparotomy, although this has been reported less often in the literature. The pathogenesis of PCI remains to be explored involving four major hypotheses to be discussed [5, 21, 36]: (a) The mechanical theory; increased intraluminal colonic gas and pressure due to fever or ileus caused impaired structural integrity of the intestinal wall, leading to relative ischemia on the mesocolon side of the lumen allowing leakage of air. (b) The pulmonary theory; pulmonary alveolar rupture induced by chronic pulmonary diseases, reached the bowel wall along the aorta and mesenteric vessels. (c) The bacterial theory; immunity was reduced by chemotherapy medications, and aerogenic bacteria penetrated the mucosal barrier via the relative ischemic zone producing gas within the bowel wall. (d) The chemical theory or nutritional deficiency theory; the toxic effects of chemotherapy on cell proliferation and apoptosis led to a compromised self-healing function of the intestinal mucosa. Malnutrition, which frequently occurs in patients post chemotherapy, increases bacterial fermentation by interfering with the consumption of carbohydrates, causing a subsequent accumulation of gas and aggravated damage to the bowel walls.

To search for the specific causative agent in our case, a literature review was conducted focused on PI/PCI post chemotherapy without allo-HSCT in hematological malignancies, which indicated the possible pathogenicity of etoposide. As a broad-spectrum chemotherapeutic agent that negatively targets topoisomerase II, and interferes with the restoration of impaired DNA [37], etoposide has been approved for clinical use by the Food and Drug Administration (FDA) of the USA in small lung cell cancer, reproductive system tumors, hematological malignancies, etc. [38, 39]. PI/PCI after etoposide-based chemotherapy has been observed in patients with lymphomas, acute myeloid leukemia, small cell lung cancer and breast cancer (Table 2). A potential mechanism was proposed by PK Bhamidipati1 et al. [24]., stating that chemotherapy and immunosuppression might result in atrophy of Peyer’s patches, leading to loss of intestinal mucosa integrity and inducing intestinal infection or gas migration. In addition, pneumoperitoneum after chemotherapy could be a manifestation of neutropenic enterocolitis (NE) complicated with bowel perforation [40], which could aggravate mucosal damage and intestinal infection. While suspected tislelizumab-induced PCI should not be excluded, albeit no previous description has been published. Temporary agranulocytosis was observed in our patient, conferring a risk of infection. The gastrointestinal tract is known to be a commonly affected system, and enterocolitis induced by immune checkpoint inhibitors (ICIs) is considered to be a secondary cause of IBD [41], which is more frequently reported in cytotoxic T-lymphocyte-associated protein-4 (CTLA-4) than PD-1 checkpoint blockade [42, 43]. Moreover, the patient’s bowel movement was normal during the week after injection of tislelizumab, and the patient presented with constipation instead of diarrhea in the early stage of onset.

Concerning the treatments for PI, conservative treatments, including oxygen therapy, antibiotics and parenteral nutrition, are recommended for individuals with apparent manifestations [8]. Compared to plain radiography, CT is more sensitive in the accurate diagnosis of PI with benefits in identifying linear gas bubbles as potential life-threatening situations [44]. PI complicated with bowel obstruction or ischemia tends to require emergency surgical intervention, which could be associated with a higher clinical severity score (including degrees of pain, diarrhea, fever, tenderness, blood per rectum, and hypotension) and metabolic acidosis (particularly increased lactic acid and significantly elevated serum amylase) [45]. Recently, a retrospective study indicated that surgical intervention was more frequently performed in the geriatric group (age ≥ 60 years) with WBC > 12 × 10^9^/L and/or emesis, while sepsis might be a risk factor for death [46]. Moreover, IAP was monitored in our case to consider its role as an alternative predictor of surgical intervention (Fig. 4). According to expert consensus [47], intra-abdominal hypertension (IAH) was defined as a sustained elevation of IAP > 12 mmHg (> 10 mmHg for children), and a decompressive laparotomy is recommended only in cases of overt abdominal compartment syndrome (ACS) recognized as IAP > 20 mmHg. Although sequential organ failure was seldom reported in patients with PI, the elevated IAP indicated a potential burden of ischemia due to excess gas trapped in the intestinal wall when surgical intervention should be considered. Compared to laparotomy, diagnostic laparoscopy was recommended as a priority for patients presenting with a threatened bowel and/or compromised bowel without significant bowel distension [48].

To our knowledge, this is the first review focused on etoposide-related PCI. Furthermore, the possibility of IAP as a predictor for surgical intervention was proposed innovatively. We shared this case with the hope of raising a clinical awareness of PCI associated with etoposide, while whether PCI could be identified as an adverse event caused by etoposide requires more evidence.

## Data Availability

The clinical information of the patient and materials generated during the current study are included within the article.

## Abbreviations

PCI: Pneumatosis cystoides intestinalis
PI: Pneumatosis intestinalis
CT: Computed tomography
IBD: Inflammatory bowel diseases
HIV: Human immunodeficiency virus
COPD: Chronic obstructive pulmonary disease
allo-HSCT: Allogeneic hematopoietic stem cell transplantation
α-GI: Alpha-glucosidase inhibitors
CAEBV: Chronic active Epstein-Barr virus
EBV-LPD: EBV-lymphoproliferative disease
HPS: Hemophagocytic syndrome
biw: Biweekly
qd: Quaque die
PD-1: Programmed cell death-1
CRS: Cytokine release syndrome
T: Temperature
P: Pulse
R: Respiration
bpm: Beats per minute
BP: Blood pressure
SpO_2_: Pulse oximetry saturation
pH: Potential of hydrogen
PaO_2_: Partial pressure of Oxygen
PaCO_2_: Partial pressure of carbon dioxide
HCO_3_: Bicarbonate
CD: Cluster of differentiation
HGB: Hemoglobin
WBC: White blood cell
PLT: Platelet
IL-2R: Interleukin-2 receptor
AST: Glutamic oxaloacetic transaminase
ALT: Glutamic pyruvic transaminase
ALP: Alkaline phosphatase
LDH: Lactic dehydrogenase
ARDS: Acute respiratory failure syndrome
IAP: Intra-abdominal pressure
IAH: Intra-abdominal hypertension
GVHD: Digestive graft versus host diseases
NE: Neutropenic enterocolitis
CTLA-4: Cytotoxic T-lymphocyte-associated protein-4
ACS: Abdominal compartment syndrome
